# Attitudes to acute pain and the use of pain assessment scales among Spanish small animal veterinarians

**DOI:** 10.3389/fvets.2023.1302528

**Published:** 2023-12-18

**Authors:** Sandra Menéndez, Miguel Angel Cabezas, Ignacio A. Gomez de Segura

**Affiliations:** ^1^Department of Animal Medicine and Surgery, University Complutense, Madrid, Spain; ^2^Dolorvet, Anestesia y analgesia veterinaria, Madrid, Spain

**Keywords:** acute pain, assessment, behavioral-based pain scales, Glasgow composite measure pain scale, feline grimace scale, dogs, cats, survey

## Abstract

Evaluation of acute pain in dogs and cats is the basis for an appropriate treatment and improved well-being. The difficulties involved in pain assessment pose a challenge for veterinarians, and recent findings indicate that the most effective tools available—validated pain assessment scales—may not be widely utilized.

**Objectives:**

This study aimed to characterize the level of concern among Spanish veterinarians regarding acute pain in companion animals. Additionally, it sought to determine whether this concern correlates with the utilization of optimal assessment tools.

**Methods:**

A survey was conducted to explore Spanish veterinarians’ attitudes toward pain and its assessment. The survey was distributed through two most prominent small animal veterinary associations, the Spanish association for veterinary anesthesia and analgesia, as well as key industry players committed to proactive pain management. Descriptive analysis of the collected data was performed using Excel and SPSS.

**Results:**

A total of 292 veterinarians participated in the study. A high level of concern regarding pain in dogs and cats was determined where 44% of surveyed veterinarians assessed pain in all patients. Despite an awareness of validated pain scales, only 28% used them. The preferred scales were the Glasgow CMPS for dogs (94%) and the Feline Grimace Scale for cats (93%). Among respondents who do not use these validated tools, there was a considerable interest in incorporating these scales into practice (85%) and considered lack of training was the most relevant issue (32%). Other challenges to scale utilization were identified, including constraints related to time, staffing, and the need to establish a habit.

**Conclusions and relevance:**

Spanish small animal veterinarians demonstrated a strong awareness to pain in their patients and employed various methods for pain assessment. However, a limited use of validated tools was identified and likely attributed to challenges such as a lack of established routine, time constraints, insufficient personnel, and, notably, a knowledge gap among veterinarians who do not employ pain assessment scales. The most commonly used scales were the Glasgow CMPS for dogs and the FGS for cats. Overall, these results suggest a window of opportunity for the implementation of training programs in small animal pain assessment at a national level.

## Introduction

The recognition and assessment of pain stand as essential requisites for optimizing the health and well-being of patients (1). Failure to recognize pain not only poses a welfare concern but also leads to undesirable physiological consequences, including activation of the sympathetic nervous system, immunosuppression, altered metabolism, impaired healing, increased morbidity, and effects on disease progression, among others. Besides, neural processes activated by pain such as sensitization may develop (2). Pain assessment allows optimal analgesic treatment thus preventing the physiological consequences that pain perception causes (e.g., cardiovascular, and behavioral alterations). Neural processes can also be prevented by promptly recognizing pain (e.g., sensitization, changes in threshold activation). Moreover, when addressing surgical pain, a correct pain assessment improves recovery time and diminishes possible complications during the post-surgical period. Acknowledged as the fourth vital sign, pain assessment must be an integral part of every physical examination (2). Heightened awareness regarding the significance of pain has led to relevant advancements, with various guidelines outlining pain assessment methodologies accessible to veterinarians globally (2–4).

The lack of direct communication with veterinary patients requires a proxy and the use of validated scales for the assessment of pain. However, the lack of adoption of validated pain scales introduces biases into veterinarians’ pain evaluations. Feline pain has frequently been underestimated, and within comparable surgical procedures performed on both dogs and cats, cats have received lesser analgesia, potentially due to the more subtle manifestation of pain signs in felines (4). Consequently, pain assessment should strive for objectivity, employing validated and species-specific tools, especially concerning cats (2). These tools are grounded in the observation of behaviors, such as the Glasgow Composite Measures Pain Scale (CMPS) for dogs and cats or the UNESP-Botucatu Feline Multidimensional Pain Assessment Scale - shortened version (UFEPS-SF) for cats, and also in cats facial expressions (Glasgow Feline CMPS scale, Feline Grimace Scale, FGS).

The importance attributed to pain by veterinarians has been on the rise (5), with many of them using pain scores recently introduced into clinical practice (2). Nonetheless, a substantial proportion of veterinarians still do not routinely incorporate validated pain assessment tools (6). While some may feel confident in their ability to detect, assess, and manage pain to a certain degree (6–8), evidence underscores the suboptimal nature of pain assessment (9). Veterinarians acknowledge the requirement for additional training in pain management (6), suggesting a gap exists between the development and dissemination of validated pain assessment tools and their routine utilization in clinical practice (2). Besides pain scales, health-related quality of life tools have been introduced to provide a more comprehensive approach to the welfare of the animals, which usually involve pain assessment as one of the most relevant factors affecting it (10).

This study’s objective was to delineate attitudes toward acute pain in dogs and cats among Spanish veterinarians. More specifically and considering the relevance of the use of the most appropriate pain assessment tools, we aimed to evaluate their knowledge among veterinarians and the attitudes to its use, as well as the impediments hampering their application. Knowledge of the veterinarian’s attitudes allows to determine whether they should be improved and, more specifically, serve as a foundation for designing strategies to enhance the widespread adoption of these tools among Spanish veterinarians.

## Materials and methods

### Survey

An online questionnaire was formulated (see Appendix) with the objective of evaluating the attitudes of Spanish small animal veterinarians regarding acute pain, whatever the cause, in dogs and cats, as well as their utilization of assessment methods, specifically pain assessment scales. The survey’s design followed the CHERRIES guidelines (The Checklist for Reporting Results of Internet E-Surveys) and was endorsed by the Institutional Ethics Committee (ref. CE_20221215-18_SAL).

The survey was structured into four sections, offering respondents the choice of single or multiple-choice answers, including open-ended text responses. Participants could skip questions as they progressed through the survey. Questions involving graded responses adhered to a consistent sequence to facilitate respondents’ input, spanning from minimum to maximum.

The survey commenced with an introduction detailing its purpose, followed by three primary sections and a concluding section featuring summary queries. The initial segment gathered demographic data to describe the profile and background of the veterinarians. The second section aimed to outline the veterinarian’s attitudes toward acute pain, encompassing parameters like which patients are assessed, the timing and methodology for pain evaluation, and whether pain assessment scales are employed. The knowledge of pain scales directed respondents to distinct questions in the third segment, using adaptive questions to minimize respondent fatigue and enhance survey completion (11). The third and final section included queries about the use of pain assessment scales and identified hindrances to their implementation. This section varied based on the prior use of pain scales. Lastly, all participants could respond to wrap-up questions concerning post-discharge follow-up practices, familiarity with pain assessment applications/websites, and an open-ended question for additional input. The questions were transferred to an online platform (Google Forms) to easy accessibility and streamline data collection. The online format enabled veterinarians to provide responses via electronic devices while ensuring anonymity. To guarantee confidentiality, the Google form gathered anonymous data, with participants being duly informed. To prevent multiple responses from a single user, access was granted via email registration (11). A pilot test was conducted before the final survey distribution to ensure coherence and clarity, with adjustments made as necessary.

### Survey distribution

The survey was aimed at veterinarians actively practicing or who have practiced small animal clinical work. Distribution occurred via email through the Spanish small animal veterinary association, which boasts 5,600 members (AVEPA^1^), the largest and most representative regional association (Madrid, AMVAC), and the Spanish Society of Veterinary Anesthesia and Analgesia (SEAAV). Additionally, distribution was facilitated through the databases of two pertinent veterinary pharmaceutical laboratories (B. Braun and Zoetis). The survey was accompanied by an introduction clarifying its purpose, respondent anonymity, and the estimated time for completion (5–10 min). The survey remained accessible from March 1, 2023, to May 12, 2023.

### Data analysis

For descriptive statistical analysis, responses from the forms were exported to Microsoft Excel. Duplicate responses, identified by identical reference email addresses, were excluded. A structured database was formulated. Descriptive analysis (IBM SPSS Statistics 28.0) was employed to render frequency and percentage representations of the variables across the entire population. Given the variations in response counts for each question, results were presented as the number of respondents selecting each answer and the percentage relative to the total responses for each question. The relationship between respondents’ knowledge and use of pain scales and their self-reported specialization was assessed using the Chi-Squared test. To gage the margin of error, considering the number of responses, an online margin of error calculator was utilized.^2^

## Results

### Demographic data

A total of 292 respondents participated in the survey. Considering Spain’s professional veterinary population, which numbered 36,337 veterinarians as of December 2022, with an estimated 60.2% (21,875 veterinarians) engaged in small animal practice (12), a margin of error of 6% was considered for the results (95% confidence level). For questions with fewer respondents (82), the margin of error was set at 11%.

Among the respondents, age distribution was relatively even, with 42% (*n* = 292) falling under the age of 40, while the remaining 58% were over 40 years old. Most were female (73%). Although participants hailed from various regions across Spain (17 Autonomous Communities), responses were more pronounced in densely populated areas. Madrid accounted for 24%, Catalonia for 17%, followed by Andalusia (12%) and the Valencia region (10%).

Regarding specialization, 27% of respondents did not consider themselves being specialized in any clinical area. Among those who did, 21% claimed expertise in internal medicine, while 14% specialized in surgery and an equal percentage in anesthesia and analgesia. Notably, 66% lacked specialization accreditation. For accredited veterinarians, 19% (*n* = 292) had completed postgraduate courses, 8% held the AVEPA accreditation, 4% had accomplished a master’s program, 3% possessed European or American diplomas, and the remaining held supplementary qualifications (IVAS diploma, internship, PhD, European college residency).

The most prevalent types of centers among respondents were practices with surgical facilities (45%) and those providing emergency services (26%). Most centers employed 2–3 veterinarians (43%; *n* = 291), and the most common employment statuses were either being employed by the center (48%; *n* = 290) or serving as a practice owner/partner (43%).

### Attitudes toward pain

**Figure 1:**
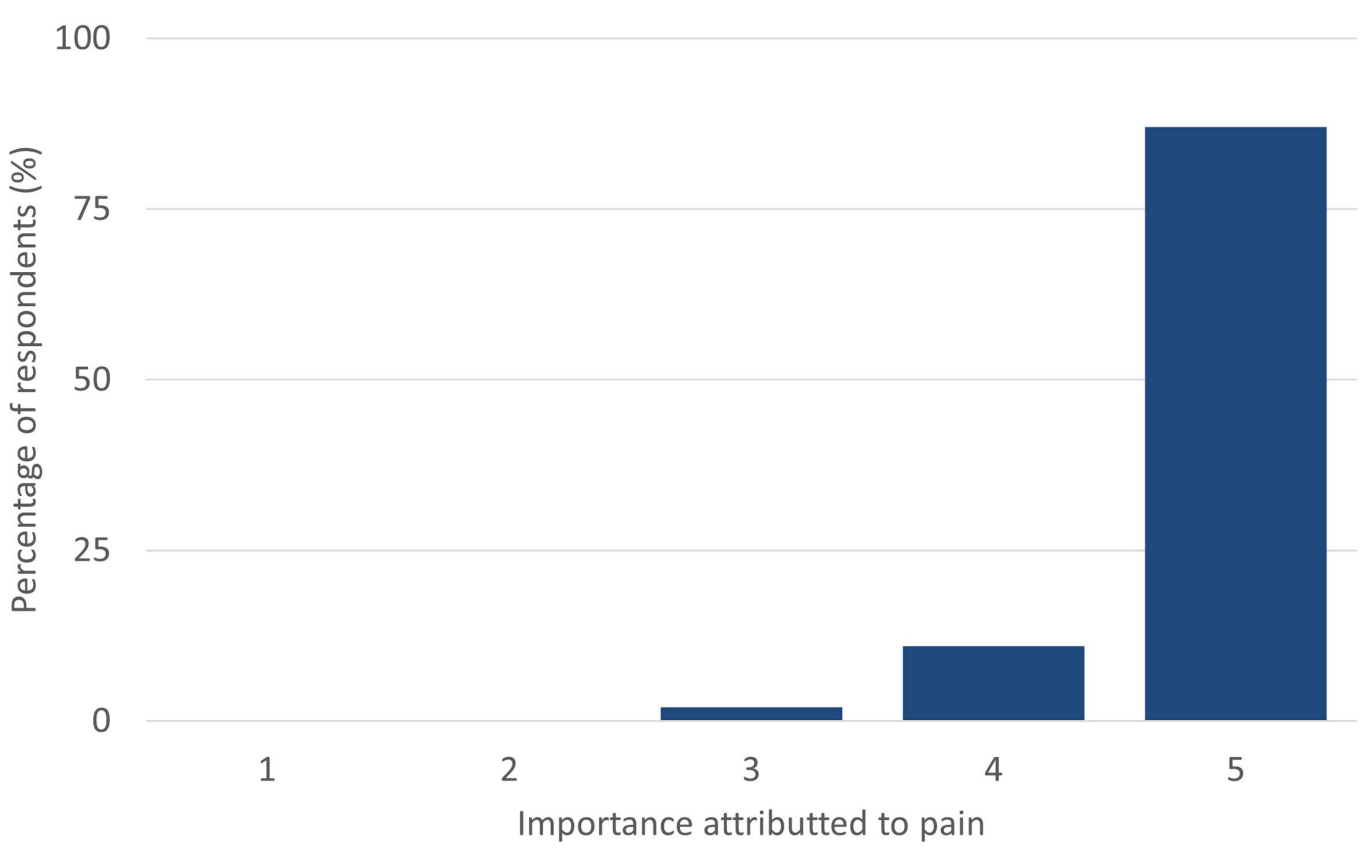
Importance attributed to pain by online surveyed small animal Spanish veterinarians in 2023 (5 indicates maximum importance, 0 indicates no importance; *n* = 292).

Most respondents (87%; *n* = 292) rated pain in their patients as highly relevant (5/5), with none attributing a relevance level of 1 (none) or 2 (low) out of 5 (Figure 1). For 71% of respondents (*n* = 292), pain severity was similar between dogs and cats when considering the same cause. In 12% of instances, dogs were perceived to experience more pain, compared to 6% for cats. The remaining 11% were uncertain. When asked about the relative difficulty of assessing pain across species, 87% (*n* = 292) considered it more challenging in cats.

### Pain assessment

**Figure 2:**
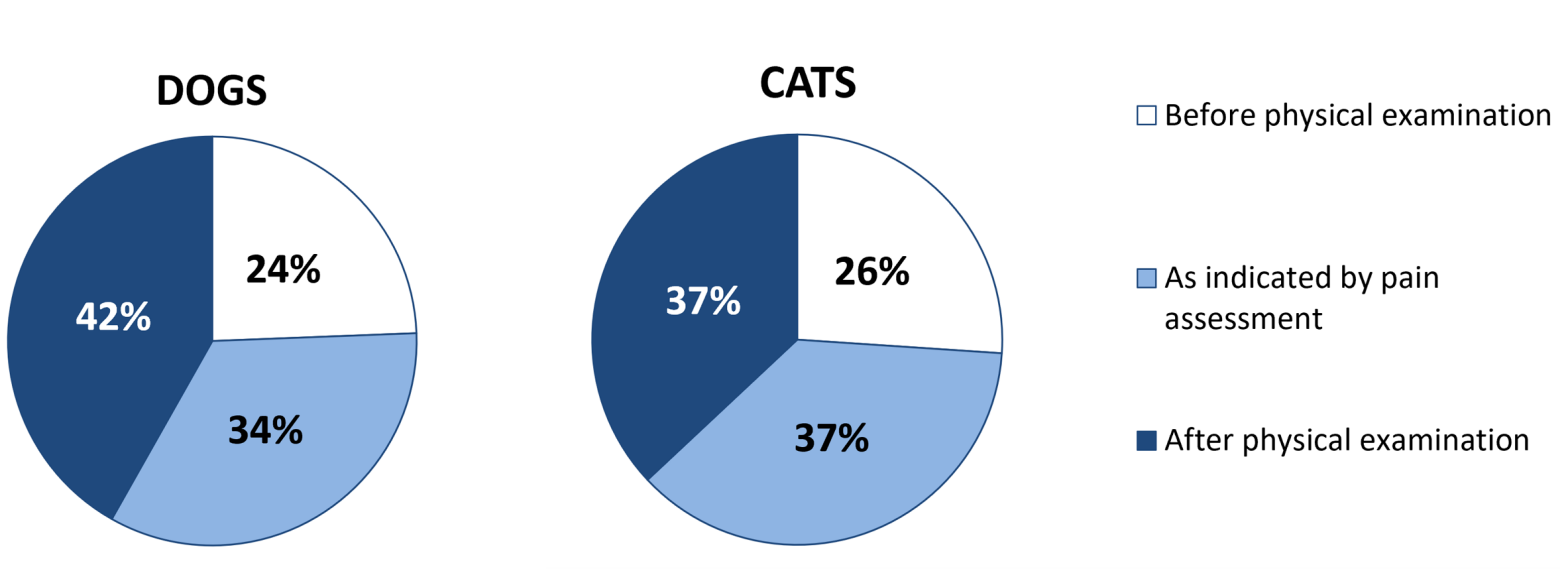
Timing of pain assessment in dogs (*n* = 287) and cats (*n* = 284) performed by online surveyed small animal Spanish veterinarians in 2023.

Most respondents reported they were assessing pain in all patients while performing physical exams (44%; *n* = 290) or when signs of pain were identified (43%). Within hospitalized and postoperative patients, only 26% of respondents (*n* = 290) reported to conduct pain assessments for all such cases. Thus, 85% (*n* = 290) consistently assessed pain, either always or at the slightest sign suggestive of mild pain. An additional 14% of respondents (*n* = 290) initiated assessments when patients were considered to be with moderate pain. This chosen timing of assessment exhibited an even distribution and similarity between dogs and cats (Figure 2).

In postoperative and/or hospitalized patients, the reported most common frequency of pain assessments was every 4–6 h, particularly during the initial stages (45%; *n* = 288), or as part of routine assessments (30%). A low percentage of respondents (6%) stated they never assessed pain in such patients.

### Assessment of analgesic efficacy

Upon administering additional doses of analgesic drugs, 50% (*n* = 289) of respondents assessed pain thereafter within 30–60 min to gage their efficacy. Moreover, 42% assessed pain during regular patient evaluations. Only 2% refrained from assessing pain after administering extra doses, citing a lack of necessity.

### Pain assessment scales usage

**Figure 3:**
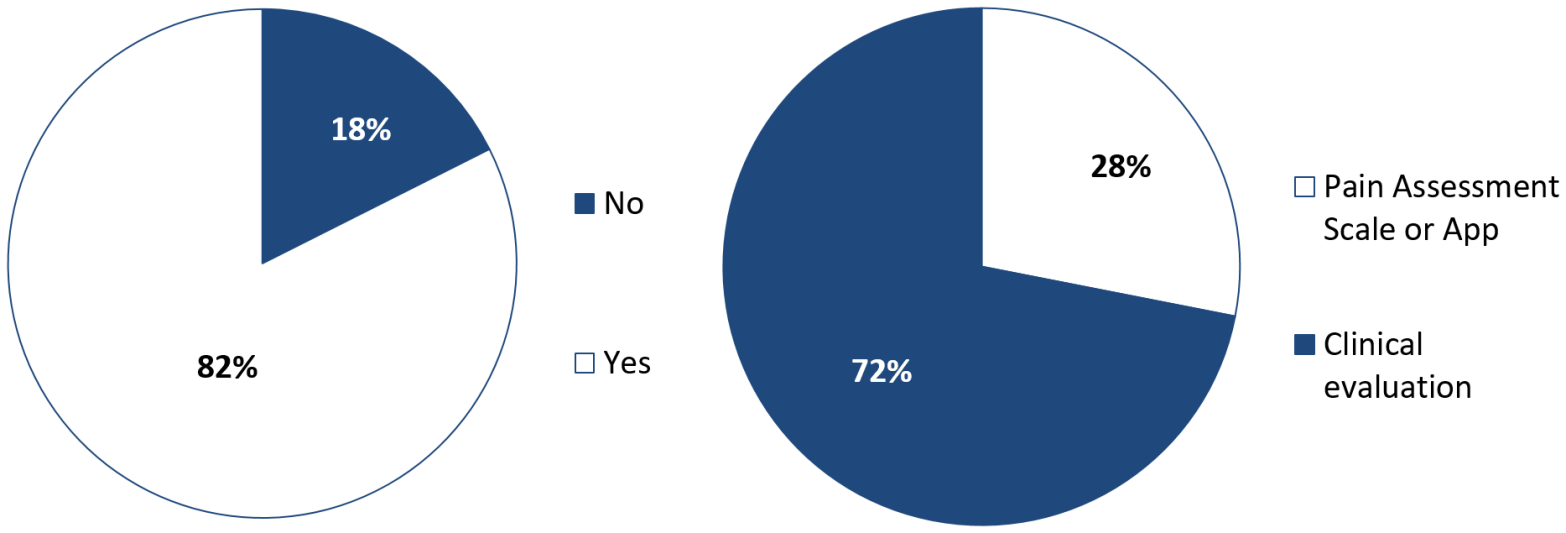
Awareness of pain assessment scales (left), and pain assessment methods used (right), among online surveyed small animal Spanish veterinarians in 2023 (*n* = 290).

A total of 239 veterinarians (82%, *n* = 292) reported being acquainted with pain assessment scales (Figure 3). Respondents who self-reported specialization in anesthesia and analgesia exhibited a significantly higher knowledge and use of pain scales (Pearson’s Chi-Squared; *p*-values: 0.030 and <0.001, respectively). Among all respondents self-reporting specialization in any veterinary specialty, a higher use of pain scales was observed (Pearson’s Chi-Squared; *p*-values: 0.006), while knowledge of pain scales did not differ when compared to respondents self-reporting no specialization.

#### Veterinarians using pain assessment scales

Among the surveyed veterinarians, 28% (*n* = 292) reported the use of pain assessment scales. Of these, 59% (*n* = 82) assigned these scales the highest utility rating on a scale of 1 to 5, where 1 represents minimal usefulness and 5 the maximum. None of the respondents assigned the lowest values of 1 or 2, and only 6% assigned a value of 3. Most respondents reported frequent (46%; *n* = 80) or routine (45%) use of these scales.

**Figure 4:**
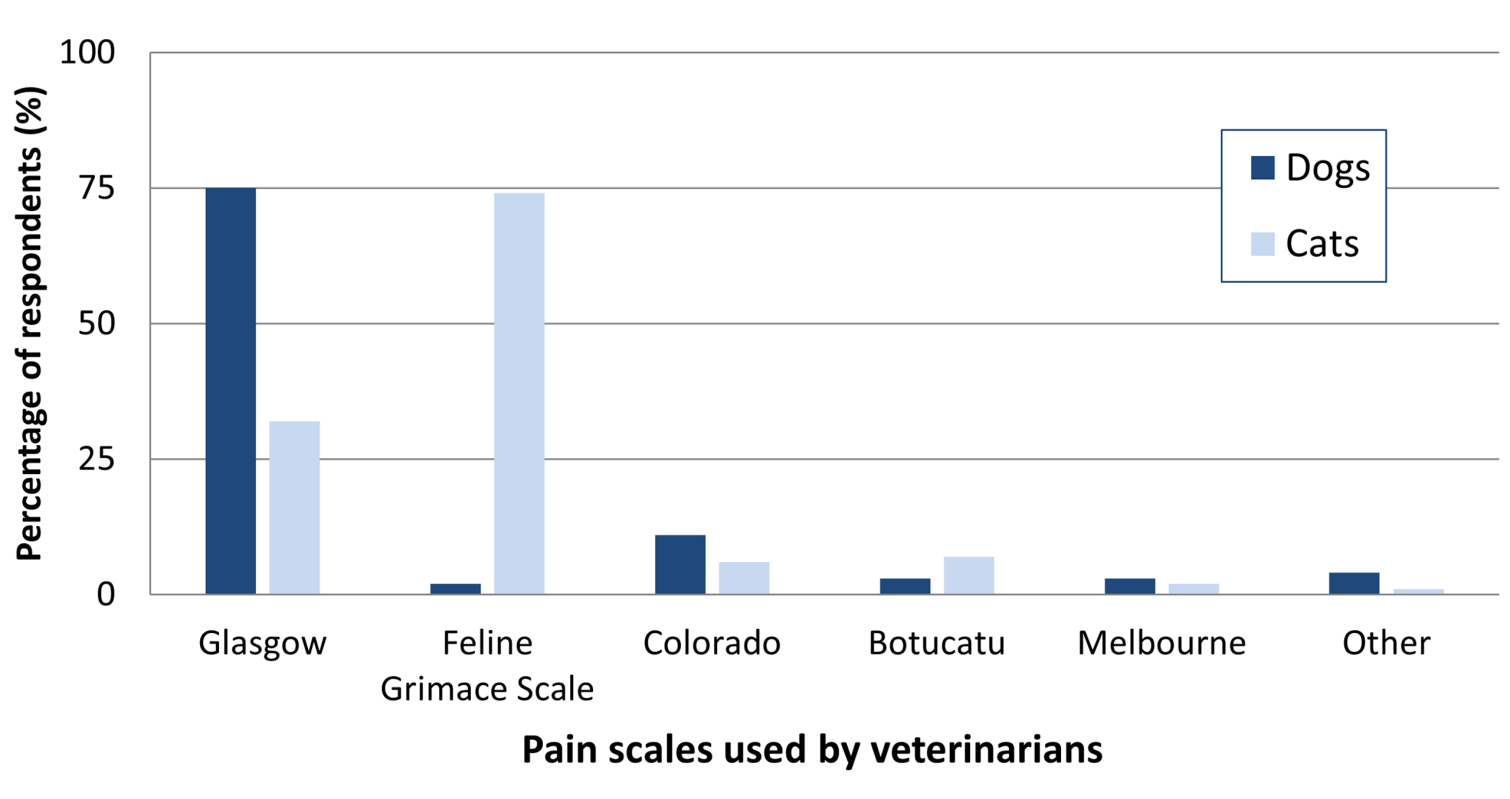
Pain assessment scales used by online surveyed small animal Spanish veterinarians in 2023 (responses from those who use scales are included, *n* = 80).

The most frequently employed pain assessment scale for dogs was the Glasgow CMPS and its shortened version (94%; *n* = 75). In contrast, for cats, the Facial Expression Scale (Feline Grimace Scale; FGS) was predominantly used (93%; *n* = 74). Anecdotally, 2 veterinarians reported using the FGS for dogs, and 3 veterinarians the UFEPS-SF for dogs, although these scales are specific to cats (Figure 4).

**Figure 5:**
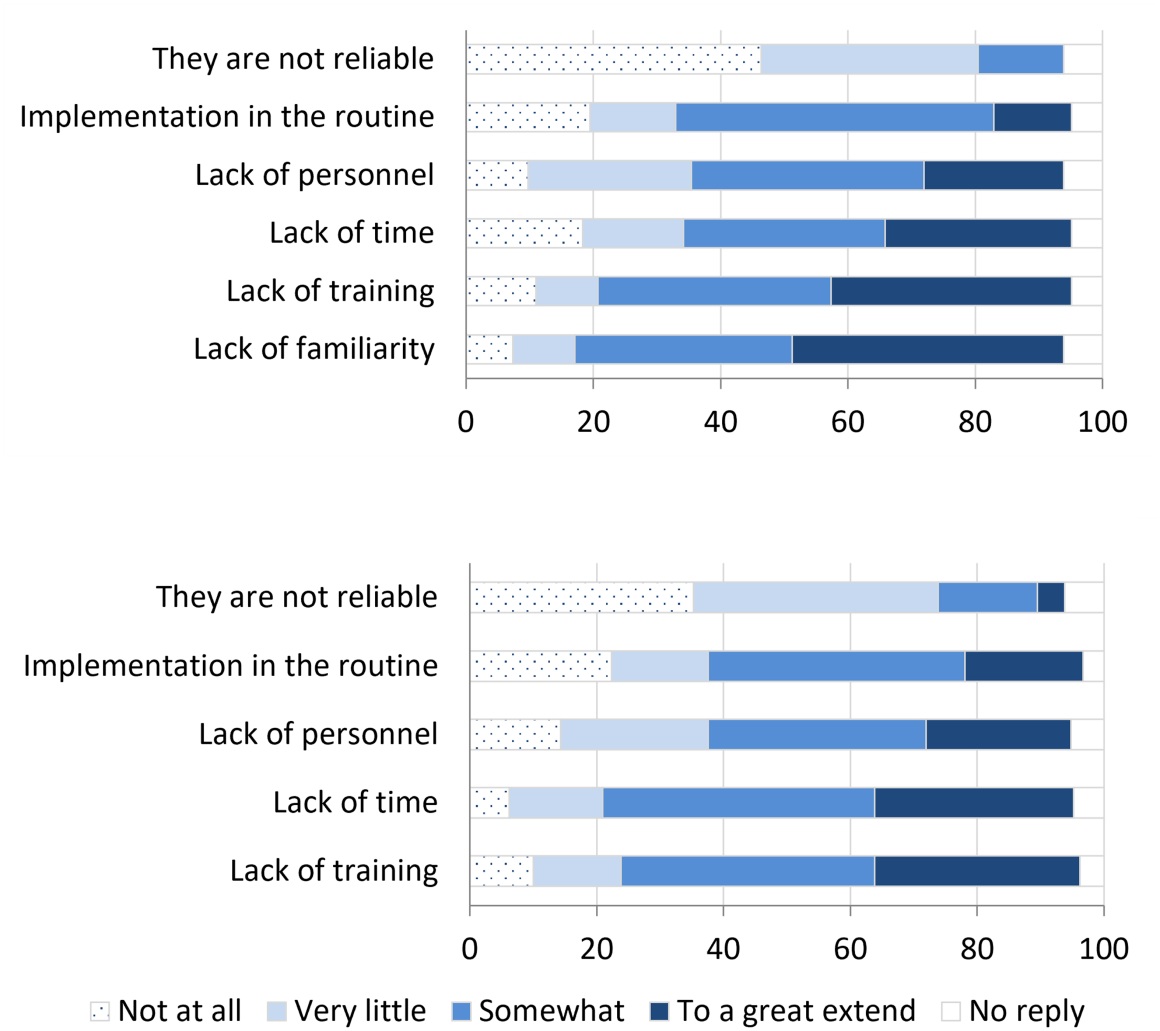
Limiting Factors for the Use of Pain Assessment Scales by online surveyed small animal Spanish veterinarians in 2023. Top: Among Veterinarians Using Scales (*n* = 82). Bottom: Among Veterinarians Not Using Scales (*n* = 210).

When examining the factors that could hinder the use of pain assessment scales, based on the input from veterinarians who utilize them, the most cited limiting factors were lack of familiarity, time constraints, and insufficient personnel. These were followed by perceived inadequacy of training, which most respondents believed somewhat hampers the utilization of these scales, alongside their integration into routine practice. Reliability was generally not considered a limiting factor (Figure 5).

Veterinarians not using pain assessment scales 72% of respondents did not employ these scales, instead relying on clinical evaluation Among them, 85% expressed interest in incorporating pain assessment scales into their practice. The primary challenges they identified that might constrain their adoption of these scales were insufficient training and time constraints. To a lesser degree, veterinarians also cited absence of clinical routine and limited personnel as constraining factors. In this context, a minority of respondents (4%, *n* = 210) believed that the low reliability of the pain scales limited their use. However, most respondents believed that the reliability of the scales either facilitated their use or had only a slight hindrance (Figure 5).

### Follow-up practices

In terms of patient follow-up practices, 58% (*n* = 291) scheduled revision consultations, and 35% recommended owners to contact them if they believed the patient was in pain. A total of 3% did not suggest follow-up or considered it unnecessary.

### Resources

Awareness of available software applications (apps and websites) among the respondents was low, with 62% (*n* = 291) not being familiar with any. Among those who were aware, 35% knew the “Feline Grimace Scale (FGS)” app, 13% the “B. Braun Te ayuda” website. Only one respondent was aware of the “PainVET” app.

## Discussion

The survey provides relevant insights into the attitudes of Spanish small animal veterinarians to pain and its assessment. Overall, they place a high relevance on identifying pain in both dogs and cats where assessment is a widely adopted practice. Respondents commonly found it more challenging to evaluate pain in cats, which could contribute to less effective pain management for felines. Most veterinarians performed pain assessments for all patients and maintained appropriate intervals for assessing pain in hospitalized, post-surgical, and medicated animals. However, despite the awareness of available and validated pain assessment scales, their usage remains uncommon among veterinarians. The factors hindering their adoption include a lack of established practice routine, time constraints, and limited personnel. Lack of training is a key reason cited by veterinarians who do not use these scales.

The gathered responses are reasonable representation of small animal veterinarians in Spain (13) and provide a description of their attitudes toward pain. As in other opinion surveys (1, 6–8), female respondents predominate over males and is consistent with the proportion of female veterinarians in Spain (12). Regional responses, higher from Madrid and Catalonia areas, reflect the higher number of small animal practices (13). Most respondents acknowledged some degree of specialization in internal medicine, surgery, and anesthesia and analgesia, likely due to their higher involvement in pain management, and mostly practicing in medium size small animal veterinary practices (69%).

Veterinarians are increasingly concerned about pain (7, 8, 14–16) moving away from the belief that pain could be useful in post-surgical patients, which was held by veterinarians some 20 years earlier in countries such as the UK (17) or Finland (18). Most respondents considered that dogs and cats experience pain to a similar extend (71%) although the administration of analgesics to cats might be lower than to dogs, likely because of the relative difficulty veterinarians might have in recognizing and evaluating pain in cats (8, 16). Indeed, this does not mean that cats perceive less pain than dogs simply because humans may not be able to recognize or assess their pain in the same way. Not all species show obvious signs of pain and those who does may differ among them. This might explain why relying on clinical assessment methods may underscore pain in species such as felines. Pain should be considered the fourth vital sign to assess, following body temperature, pulse, and respiratory rate (2, 3), and is consistent with the high rate of veterinarians who assess pain in all patients.

Most surveyed veterinarians (85%) reported to assess pain in their patients, either always or at the slightest indication of mild pain. However, when asked if they were assessing pain in postoperative patients routinely, less than one-half of respondents (44%) assessed pain in all patients. Such discrepancy may reflect the reliance on the clinical assessment of pain and the recognition of signs of pain. However, standard clinical pain assessment lacks necessary validation, and there is no singular sign of pain in animals—be it behavioral, physiological, or endocrine—raising concerns about its effectiveness for ensuring adequate pain management. Given that the success of an analgesic protocol relies significantly on an accurate pain assessment, we strongly advocate for the widespread use of validated pain scales among clinicians, thus preventing or largely reducing pain-related alterations (2). After surgery, veterinarians initially assessed pain more frequently, and then subsequently every 4–6 h or during routine evaluations. The frequency of assessment depends on each patient’s pain intensity and can be done every 2–4 h, also depending on the analgesic drugs used (2).

It is surprising that 6% of veterinarians in this study reported not evaluating pain in hospitalized or postoperative patients and suggest further training in pain management is still required. A similar rate has also been observed among Swiss veterinarians (19). Most responses reported the evaluation of the effectiveness of additional analgesic doses at 30–60 min. Re-evaluation should ideally start from 30 min after administering analgesic medications (9).

There is an increasing knowledge of pain assessment tools among veterinarians. In 2004, only 27% of French veterinarians were aware of pain scales (14), while 82% of Spanish respondents acknowledged them in the present survey. Such increase is likely the result of the development and clinical dissemination of such scales over the last years (20). As expected, respondents self-reporting specialization were more likely to use pain scales, particularly those specialized in anesthesia and analgesia. These findings suggest that training in pain assessment should be targeted primarily at non-specialized veterinarians or the general practitioner.

Among pain scales, multidimensional pain scales have been developed improving pain assessment and management, where validated scales are preferred (20–23). However, its use is suboptimal (1, 6), with only 28% of Spanish respondents reporting their use. Previous studies reported a 10% usage from Canadian practitioners (24), or 20% from Australian practitioners (25), but recent evidence among US practitioners indicate the use of pain scoring close to 50% (5). These data strongly suggest an increased concern from practitioners on the use of more reliable pain assessment methods (24). Lack of training was perceived the main limiting factor, followed by a lack of time and personnel, to the use of validated scales by respondents not already using them. These factors are commonly reported in similar surveys (5, 24, 26, 27). The willingness of most veterinarians not using pain assessment scales (85%) to consider them in their practice should be considered an opportunity to establish appropriate training programs (23). Duration required for completion of the pain scales has been another perceived limiting factor (5). However, the Glasgow CMPS in its shortened form (CMPS-SF) only requires 2 min, even less with sufficient experience (28). Another perceived limiting factor was the anticipated difficulty to the integration of these scales into the practice’s routine together with a lack of compliance (5, 28).

The usefulness of pain assessment scales is highly regarded by those respondents who use them, mostly always or frequently. In a survey among veterinarians in the USA, nearly half of the respondents (48%) reported the routine use of pain scales after surgery or in painful procedures, with an additional 16% of respondents using them sometimes (5). These results suggest that once veterinarians become familiar with the scales, they are inclined to use them regularly, although this likely includes the more motivated or pain-aware veterinarians. The clinical introduction of pain scales may be facilitated by scales with a threshold for administering analgesics or modifying current therapy to reduce pain. The Glasgow CMPS is an example, with a threshold of 6 out of 24 maximum points guiding patient therapy (21).

The most widely used pain assessment scale for dogs by Spanish respondents was the Glasgow CMPS (94%) followed, to a much lesser extent, by the Colorado state university pain scale (14%). This figure is higher than the previously reported use of the Glasgow CMPS scale (44%) (1) but suggest the widespread use of this scale, supported by scientific backing, validation, and availability in seven languages, including Spanish, making it suitable for different geographical locations (2). Interestingly, in the USA the, non-validated, Colorado state university pain scale was considered the best tool by 37% of the surveyed population, followed by the numerical rating scale (17%) and the Glasgow CMPS (12%) (5), likely reflecting geographical differences. In cats, the most used scale among respondents was the Feline Grimace Scale (FGS), followed by the Glasgow Feline CMPS, and the University of São Paulo Multidimensional Feline Pain Scale in its abbreviated form (UFEPS-SF). The FGS, based on facial expressions, can be applied to any type of acute pain; it is a validated and easily interpretable tool that does not require direct interaction and has a cut-off value, potentially contributing to its favored use. The Glasgow Feline CMPS is available in English and Spanish, and the UFEPS-SF is available in Spanish and seven other languages (2). Since it was the first validated scale, the UFEPS-SF is considered the “gold standard” for scoring pain in cats, with high specificity and sensitivity rates (28). Overall, responses from the present survey indicate a preference from Spanish veterinarians using pain assessment scales for those that are validated.

Among the limitations reported for using pain assessment tools are lack of routine, time, and personnel, the two latter reported previously (5). Although improving, training to assess pain is not yet sufficient for pre-graduate veterinary education (8) and thus is perceived as one of the most limiting factors, likely higher among older veterinarians. Reliability was not a concern from respondents to the survey although this factor has been considered by US veterinarians (5) and may reflect the higher use of unidimensional pain scales, such as the numerical rating scale, associated with higher inter-observer variability (29, 30).

The attitudes of veterinarians have a direct and relevant impact on the quality of life and well-being of their patients (31). Such attitudes involve not only those related to pain and its alleviation but also common procedures such as management or handling practices. Refinements may include the provision of a calm environment (32) or the presence of the owner (33), among others, and may greatly reduce stress during veterinary practice (34). However, these practices are not routinely performed and perceived barriers to its implementation in veterinary practice may be related to constructional aspects but also time constraints (31). Although this latter perceived barrier has also been reported by Spanish veterinarians to implement pain scales, lack of training is perceived as the main factor.

Veterinarians are also relevant in providing owners with the knowledge and skills that promote their pets’ welfare at home (34). Involving pet owners in pain recognition is becoming relevant, as they will be responsible for their pets’ care after discharge. In this survey, most veterinarians schedule follow-up appointments, and one-third recommended owners to contact them if they recognize pain in their pets. This requires providing them with tools to recognize and even assess pain, and it is foreseeable that this will be the next step (4, 35). There are websites (e.g., https://animalpain.org/; https://www.metacam-painscale.co.uk/; https://bbraunteayuda.com/) and available apps (https://www.sylvester.ai/cat-owners) that adapt existing scales to online platforms, allowing both veterinarians and owners to monitor pain in dogs and cats. More than half of the veterinarians in this survey were unaware of these tools. The “Feline Grimace Scale (FGS)” app, developed by the University of Montreal, provides accurate pain scores when used by owners (35). This resource was the most recognized by Spanish veterinarians (35%). However, most resources are in English (36) and may limit its use by non-English native veterinarians (6).

It is important to note that the survey does have limitations. Sample size involved only 292 respondents although the estimated precision was between 6 and 11%, depending on the number of responses. The responses may also be influenced by the fact that those more concerned about pain were more likely to respond. Thus, pain-scoring practices may be overestimated. The distribution of the questionnaire through selected associations and companies where registration is not mandatory might introduce bias into the results. On the other side, members of the two involved associations are expected to credit the professional value of their scientific initiatives and activities. Both hold the two main scientific conferences in Spain for small animal veterinarians and distribution of the survey was previously reviewed and approved by their scientific committees. The two selected veterinary companies hold reputable positions in the veterinary field and have conducted similar activities to gage veterinarians’ attitudes toward pain.

In summary, our investigation revealed a substantial level of concern among clinicians regarding pain assessment as the optimal method for ensuring effective pain management. Clinicians are well-informed about the existence of new, straightforward, and validated tools within the clinical setting. However, there is a gap between their beliefs and its application to clinical practice. Veterinarians mostly rely on the poorly specific and sensitive clinical methods, which may lead to underscored pain and, likely, suboptimal analgesic treatment. This fact should be understood by the general practitioner to facilitate the implementation of better assessment tools. In addition, commonly perceived barriers should be of lesser importance since no additional resources are needed and the reduced time required to assess pain with most validated scales does not significantly increase the clinical burden of routine clinical assessments. Lastly, but not least, respondents already recognize the necessity for training in the utilization of these tools. Besides formal training, veterinarians may benefit from pain-related resources with online tutorials (e.g., www.animalpain.org), or validated translated tools when applicable (e.g., Spanish). This reported positive attitude to training requires the appropriate response from professional associations, urging the development of a comprehensive pain assessment training program, involving not only veterinarians but also technicians and owners.

In conclusion, Spanish small animal veterinarians express substantial concern about pain in dogs and cats and employ various methods for pain assessment. However, the use of validated pain assessment scales was limited, primarily due to factors like lack of practice routine, time, and personnel. Most veterinarians not currently using these scales express interest in adopting them, indicating a need for training. The most used scales were the Glasgow CMPS for dogs and the FGS for cats. Furthermore, as a main conclusion, further training of veterinarians is necessary to ensure an improvement in the quality of life of our patients.

## Data availability statement

The raw data supporting the conclusions of this article will be made available by the authors, without undue reservation.

## Author contributions

SM: Investigation, Methodology, Writing – review & editing. MC: Conceptualization, Investigation, Supervision, Writing – review & editing. IG: Conceptualization, Investigation, Project administration, Supervision, Writing – original draft, Writing – review & editing.

## Footnotes

^1^https://www.avepa.org

^2^https://www.surveymonkey.com/mp/margin-of-error-calculator/
