# Supplementary material for: Attitudes to acute pain and the use of pain assessment scales among Spanish small animal veterinarians
Source: Front Vet Sci. 2023 Dec 18;10:1302528. doi: 10.3389/fvets.2023.1302528 (PMC10758136; doi:10.3389/fvets.2023.1302528)
Supplement: Supplementary file 1 [file Data_Sheet_1.pdf]

1. Correo *

2. **Marca** para continuar con la encuesta

Selecciona todos los que correspondan.

- [ ] Acepto continuar con la encuesta

Datos DEMOGRÁFICOS

3. ¿Tu edad actual es...?

Marca solo un óvalo.

- Menos de 30 años.
- Entre 30 y 39 años.
- Entre 40 y 49 años.
- Más de 49 años.

4. ¿Género?

Marca solo un óvalo.

- Femenino
- Masculino
- No binario
- Prefiero no decirlo

5. ¿Cuál es tu Comunidad Autónoma?

Marca solo un óvalo.

- Andalucía
- Aragón
- Islas Baleares
- Canarias
- Cantabria
- Castilla-La Mancha
- Castilla y León
- Cataluña
- Ciudad de Ceuta
- Ciudad de Melilla
- Madrid
- Navarra
- Valenciana
- Extremadura
- Galicia
- País Vasco/Euskadi
- Principado de Asturias
- Murcia
- Rioja

6. ¿Consideras que te has especializado en alguna de las siguientes áreas?

Selecciona todos los que correspondan.

- [ ] Anestesia y Analgesia
- [ ] Cirugía
- [ ] Medicina interna (excepto oncología)
- [ ] Oncología
- [ ] Urgencias y cuidados intensivos
- [ ] Otro: ______________________

7. ¿Dispones de alguna acreditación que certifique algún grado de especialización?

Selecciona todos los que correspondan.

- [ ] Diplomado de Colegio Europeo o Americano
- [ ] Acreditado AVEPA
- [ ] No
- [ ] Otro: ____________________

8. Tipo de centro veterinario en el que trabajas (preferentemente si es más de uno):

Marca solo un óvalo.

- ◯ Clínica sin quirófano / Consultorio.
- ◯ Clínica equipada con quirófano.
- ◯ Clínica equipada con quirófano y servicio de urgencias.
- ◯ Hospital con servicio las 24 h.
- ◯ Centro de referencia.

9. Número de veterinarios que trabajan en el centro veterinario:

Marca solo un óvalo.

- ◯ 1
- ◯ 2-3
- ◯ 4-6
- ◯ Más de 6

10. En la actualidad, ¿cuál de las siguientes opciones refleja mejor tu situación laboral?

Marca solo un óvalo.

- ◯ Soy propietario o socio de una clínica de pequeños animales.
- ◯ Estoy empleado en una clínica de pequeños animales.
- ◯ Soy autónomo y trabajo para diferentes centros de pequeños animales.
- ◯ Actualmente no me dedico a la clínica de pequeños animales.

VALORACIÓN DEL DOLOR AGUDO

En esta sección pretendemos conocer como evalúas el dolor, es decir, no solo reconocer que está presente.

11. ¿Qué **importancia** consideras tiene la **evaluación del dolor agudo** en tus pacientes?

Marca solo un óvalo.

Nula

1 ◯

2 ◯

3 ◯

4 ◯

5 ◯

Máxima

12. En general, ¿Piensas que los perros y gatos sienten el **mismo grado de dolor** frente a una misma dolencia?

Marca solo un óvalo.

- ◯ Sí, o al menos muy similar.
- ◯ No, los gatos sienten más dolor que los perros.
- ◯ No, los perros sienten más dolor que los gatos.
- ◯ No lo sé.

13. ¿Cómo consideras la **dificultad relativa** de valoración del dolor en perros y gatos?

Marca solo un óvalo.

- ◯ Igual de difícil.
- ◯ Más difícil valorar en perros.
- ◯ Más difícil valorar en gatos.
- ◯ No lo sé.

14. ¿En cual de de las siguientes **situaciones te planteas la valoración del dolor**? En este caso no nos referimos al hecho de reconocer que hay dolor, sino evaluar su intensidad o características empleando criterios clínicos o algún instrumento de medida.

Selecciona todos los que correspondan.

- [ ] Lo realizo en todos los pacientes, incluidas las visitas rutinarias.
- [ ] En todos los pacientes hospitalizados y postquirúrgicos.
- [ ] Cuando identifico dolor en los pacientes
- [ ] Otro: ______________________

15. ¿Con qué **nivel mínimo de dolor** consideras su evaluación en un paciente?

Marca solo un óvalo.

- ◯ Siempre
- ◯ Dolor leve
- ◯ Dolor moderado
- ◯ Dolor severo

16. Cuando procede, ¿**Cuando valoras** el dolor en perros y gatos?

Marca solo un óvalo por fila.

	Mientras realizo la exploración del paciente (es lo primero que hago)	Mientras realizo la exploración del paciente (es lo último que hago, tras Temp., Pulso, Respiración, etc..)	A intervalos específicos según el grado de dolor del paciente
**Perro**	◯	◯	◯
**Gato**	◯	◯	◯

17. Si valoras el **dolor postoperatorio o en pacientes hospitalizados** con dolor ¿Con qué **frecuencia** lo valoras?

Marca solo un óvalo.

- Con cada valoración rutinaria del paciente.
- Más frecuente en las primeras horas y luego cada 4-6 h.
- Cada 4-6 h.
- Cada 12 h.
- Cada 24 h.
- Solo si hay signos claros de dolor.
- No es necesario, todos los pacientes reciben analgesia postoperatoria.
- Nunca
- Otro: ____________________

18. Cuando se administra una **dosis adicional,** pautada o no, de analgésicos **¿Valoras el dolor posteriormente para valorar su eficacia?**

Marca solo un óvalo.

- Si, a los 30-60 min para verificar la eficacia.
- Si, con cada valoración rutinaria del paciente.
- No, no es necesario.
- Otro: ____________________

19. ¿**Conoces las escalas** de valoración del dolor en perros o gatos?

Marca solo un óvalo.

- Si
- No

20. ¿Cómo valoras el dolor?

Marca solo un óvalo.

- Evaluación clínica (sin emplear escalas) *Salta a la pregunta 25*
- Empleando una Escala o App de evaluación del dolor

Empleo de Escalas de Evaluación del Dolor

21. ¿Qué grado de **utilidad crees que tienen las escalas de dolor** para su valoración en la clínica (1 ninguna, 5 máxima)?

Marca solo un óvalo.

	Muy baja o nula
1	◯
2	◯
3	◯
4	◯
5	◯
	Máxima

22. En pacientes con dolor ¿Con qué **frecuencia** utilizas las escalas?

Marca solo un óvalo por fila.

	Esporádicamente	Frecuentemente	Siempre
**Perro**	◯	◯	◯
**Gato**	◯	◯	◯

23. ¿**Qué escalas** de evaluación de dolor agudo **empleas**?

Selecciona todos los que correspondan.

	Ninguna	Glasgow	Botucatu	Expresión facial (Feline Grimace Scale)	Melbourne	Colorado	Otra
**Perro**	☐	☐	☐	☐	☐	☐	☐
**Gato**	☐	☐	☐	☐	☐	☐	☐

24. ¿Qué factores, y cuanto, **dificultan el empleo** de escalas de evaluación de dolor?

Marca solo un óvalo por fila.

	No lo dificultan	Poco	Algo	Mucho
**Implantarlas en la rutina del centro**	◯	◯	◯	◯
**No son muy fiables**.	◯	◯	◯	◯
**Falta de formación**.	◯	◯	◯	◯
**Falta de costumbre**.	◯	◯	◯	◯
**Falta de tiempo**.	◯	◯	◯	◯
**Falta de personal**.	◯	◯	◯	◯

Salta a la pregunta 27

Valoración Clínica

25. **Si no utilizas ninguna escala de dolor** ¿Considerarías la inclusión de alguna escala de dolor en tu centro en un **futuro próximo**?

Marca solo un óvalo.

- ◯ No es el caso, ya utilizo las escalas de dolor en todos los pacientes que lo requieren
- ◯ Sí, me gustaría empezar a usarlas
- ◯ No, no creo me ayuden mucho
- ◯ No lo sé.
- ◯ Otro: ______________________

24. ¿Qué factores, y cuanto, **dificultan el empleo** de escalas de evaluación de dolor?

Marca solo un óvalo por fila.

	No lo dificultan	Poco	Algo	Mucho
**Implantarlas en la rutina del centro**	⬭	⬭	⬭	⬭
**No son muy fiables.**	⬭	⬭	⬭	⬭
**Falta de formación.**	⬭	⬭	⬭	⬭
**Falta de costumbre.**	⬭	⬭	⬭	⬭
**Falta de tiempo.**	⬭	⬭	⬭	⬭
**Falta de personal.**	⬭	⬭	⬭	⬭

Salta a la pregunta 27

Valoración Clínica

25. **Si no utilizas ninguna escala de dolor** ¿Considerarías la inclusión de alguna escala de dolor en tu centro en un **futuro próximo**?

Marca solo un óvalo.

- ⬭ No es el caso, ya utilizo las escalas de dolor en todos los pacientes que lo requieren
- ⬭ Sí, me gustaría empezar a usarlas
- ⬭ No, no creo me ayuden mucho
- ⬭ No lo sé.
- ⬭ Otro: ______________________
